# Polymeric Nanoparticle-Mediated Photodynamic Therapy: A Synergistic Approach for Glioblastoma Treatment

**DOI:** 10.3390/ph18071057

**Published:** 2025-07-18

**Authors:** Bandar Aldhubiab, Rashed M. Almuqbil

**Affiliations:** Department of Pharmaceutical Sciences, College of Clinical Pharmacy, King Faisal University, Al-Ahsa 31982, Saudi Arabia; ralmuqbil@kfu.edu.sa

**Keywords:** glioblastoma, photodynamic therapy, polymeric nanoparticles, blood–brain barrier, clinical translation

## Abstract

Glioblastoma is the most common and aggressive malignant primary brain tumour. Patients with glioblastoma have a median survival of only around 14.6 months after diagnosis, despite the availability of various conventional multimodal treatments including chemotherapy, radiation therapy, and surgery. Therefore, photodynamic therapy (PDT) has emerged as an advanced, selective and more controlled therapeutic approach, which has minimal systemic toxicity and fewer side effects. PDT is a less invasive therapy that targets all cells or tissues that possess the photosensitizer (PS) itself, without affecting the surrounding healthy tissues. Polymeric NPs (PNPs) as carriers can improve the targeting ability and stability of PSs and co-deliver various anticancer agents to achieve combined cancer therapy. Because of their versatile tuneable features, these PNPs have the capacity to open tight junctions of the blood–brain barrier (BBB), easily transport drugs across the BBB, protect against enzymatic degradation, prolong the systemic circulation, and sustainably release the drug. Conjugated polymer NPs, poly(lactic-co-glycolic acid)-based NPs, lipid–polymer hybrid NPs, and polyethylene-glycolated PNPs have demonstrated great potential in PDT owing to their unique biocompatibility and optical properties. Although the combination of PDT and PNPs has great potential and can provide several benefits over conventional cancer therapies, there are several limitations that are hindering its translation into clinical use. This review aims to summarize the recent advances in the combined use of PNPs and PDT in the case of glioblastoma treatment. By evaluating various types of PDT and PNPs, this review emphasizes how these innovative approaches can play an important role in overcoming glioblastoma-associated critical challenges, including BBB and tumour heterogeneity. Furthermore, this review also discusses the challenges and future directions for PNPs and PDT, which provides insight into the potential solutions to various problems that are hindering their clinical translation in glioblastoma treatment.

## 1. Introduction

Glioblastoma is the most common malignant primary brain tumour, representing around 50.9% of all malignant tumours and 14.2% of all tumours [1,2]. The World Health Organization classification has designated glioblastoma as a grade IV histological malignancy [3]. In the United States, glioblastoma is the twelfth leading cause of cancer-associated deaths [4]. Glioblastoma patients have a median survival of only around 14.6 months after diagnosis, despite the availability of various conventional multimodal treatments including chemotherapy, radiation therapy, and surgery [5]. The high extent of heterogeneity within glioblastoma cells sets further hurdles in treatment, as variations in molecular characteristics and cellular structures contribute to increased drug resistance and unpredictable therapeutic outcomes [6]. Bevacizumab is used as a salvage therapy in patients with recurrent glioblastoma since its approval in 2009 [7]; however, there is no concrete proof regarding its capacity to extend overall survival [8].

Furthermore, most chemotherapeutic medications are unable to successfully cross the blood–brain barrier (BBB), therefore their overall efficacy remains low (Table 1) [9]. The BBB is composed of tightly joined endothelial cells that serve a protective function [10]; however, it poses a substantial challenge to the effective transport of chemotherapeutic agents and emerging therapies across the BBB as well as other biological barriers into the tumour, hindering glioblastoma treatment [11]. Therefore, there is a rising interest in photodynamic therapy (PDT) as an advanced, selective and more controlled therapeutic approach, coupled with minimal systemic toxicity and fewer side effects.

**Table 1:** Advantages and drawbacks of conventional therapeutic approaches in glioblastoma.

Type of Therapy	Examples	Advantages	Drawbacks	References
Surgical resection	Craniotomy	Removal of the bulk of the tumour	Impossible to remove all glioblastoma cells in a tumour; nearly all glioblastoma tumours locally recur; risk of surgical wound complications and direct cortical as well as vascular injury	[12]
Chemotherapy	Fotemustine, temozolomide, lomustine, carmustine	Slows tumour growth and reduces tumour size	Some chemotherapeutic agents cannot effectively penetrate blood–brain barrier which limits their use; fortified tumour location hinders the delivery of therapeutics; therapy resistance	[13]
Radiation therapy	Brachytherapy, 2D conventional radiotherapy, particle radiation therapy, intensity modulated radiotherapy	Usually combined with chemotherapy to treat high-grade gliomas	Radiation necrosis; normal tissues are inevitably irradiated; toxicity; cognitive dysfunction; some glioblastomas are radioresistant	[13,14]

Photodynamic therapy (PDT) is a targeted cancer treatment that uses laser light to activate a photosensitising chemical, causing reactive oxygen species (ROS) to preferentially destroy cancer cells [15]. Photodynamic therapy (PDT) can be used with chemotherapy, radiation, or various anticancer therapies to improve therapeutic efficacy by using laser-activated photosensitisers to generate reactive oxygen species (ROS) that selectively destroy cancer cells in order to tackle multidrug resistance and achieve deeper penetration in tissues via use of X-rays, two-photon excitation, or self-luminescence [16]. Although PDT offers numerous benefits in cancer treatment, it faces challenges in clinical use because of the characteristics of the PS. Most of the PS agents are hydrophobic in nature, thus showing lower water solubility and propensity to aggregate in physiological conditions, which can eventually lead to decreased efficacy of ROS generation [17]. Even after improving the water solubility of some PS drugs, their selective buildup in target cells or tissues is inadequate for successful clinical use [18]. Thus, an effective delivery system is required to overcome biological barriers in delivering the PS for the advancement of PDT [19].

The three most vital PDT components include molecular oxygen, PS, and light. Another important PDT component is a comprehensive and robust light dose; dosimetry of PS concentration needs to be carefully studied for the development of valid clinical protocols as well as outcome prediction [20]. In oxygen-saturated conditions, the rise in PS concentration and light dose typically results in a better PDT efficiency. Nonetheless, molecular signatures and additional features of tumour biology are important elements that determine success in PDT therapy against malignant tumours [21,22,23]. Various studies have already reported that a substantial level of genetic heterogeneity is present in the case of glioblastoma, even within individual tumours [24,25,26]. Furthermore, this heterogeneity results in variations in the sensitivity of tumour cells toward the anticancer agents [27,28,29,30]. Characteristics of glioblastoma cells include an increased basal ROS level and high metabolic rate that have a significant contribution as chemical mediators in the regulation of therapeutics, providing protection against malignant cells from apoptosis, and signal transduction [31].

The epidermal growth factor receptor variant III (EGFRvIII) is the most common genetic alteration observed in association with glioblastoma, therefore therapies targeting EGFRvIII have great potential in glioblastoma treatment. Amplification of EGFR is the most common genetic alteration that occurs in glioblastoma, and glioblastoma shows that amplified EGFR commonly overexpresses the receptor variant III (EGFRvIII), which indicates the importance of EGFRvIII in the case of elevated proliferation of glioma cells [32]. Glioblastoma patients with EGFRvIII-positive tumours typically show shorter survival duration. Thus, EGFRvIII has a significant influence on targeted therapy in EGFRvIII-amplified glioblastoma [33,34]. Various studies have reported numerous targeted therapeutic approaches for glioblastoma based on EGFRvIII, including anti-EGFRvIII vaccines, EGFRvIII monoclonal antibodies (MAbs), and EGFRvIII small molecule inhibitors [35,36]. Nonetheless, clinical outcomes with these therapeutics were not satisfactory. There are several factors underlying such unsatisfactory clinical outcomes including drug resistance, the presence of compensatory signalling pathways in tumour cells, glioblastoma heterogeneity, and the limited capacity of therapeutics to penetrate the BBB [37]. Thus, it is crucial to design and develop novel drugs targeting EGFRvIII [38].

Recently, checkpoint inhibitors have gained a lot of attention in cancer immunotherapy [39]. Both the suppression of cytotoxic T-lymphocyte-associated protein 4 and the inhibition of programmed cell death ligand 1, also known as PD-1, have already been licensed by the US FDA as therapies for a variety of cancer types [40,41]. Nonetheless, checkpoint inhibitors show limited efficacy in glioblastoma treatment, partially owing to the poor efficacy of drug delivery across the blood–tumour barrier (BTB) [42]. Immunosuppressive tumour microenvironment (TME) is another major challenge, which involves the expression of immunosuppressive molecules, excessive tumour-associated macrophages, and poor T cell infiltration [43,44]. Therefore, drugs including anti-PDL1 antibody (aPDL1) should be effectively delivered across the BTB in order to ameliorate the immunotherapeutic effectiveness in case of glioblastoma treatment. On the other hand, 5-aminolevulinic acid, sometimes known as 5-ALA, is a naturally occurring chemical used in PDT for gliomas because of its ability to selectively induce the generation of protoporphyrin IX (PpIX). Nonetheless, its clinical outcomes are often limited because of several factors including poor accumulation at the tumour site and low bioavailability. Thus, to overcome these limitations, there is a growing interest in incorporating 5-ALA into nanoparticles (NPs) [45].

NPs such as polymeric NPs [46], lipid-based NPs [47], liposomes [48], niosomes [49,50], nanosuspension [51], dendrimers [52], metallic NPs [53], carbon-based NPs [54,55], magnetic NPs [56], quantum dots [57], silica NPs [58], nanoemulsions [59,60], etc., have demonstrated significant potential in refining the effectiveness of drugs in various disease conditions. They also perform a significant role in PDT [61]. NPs as active agents or carriers can improve the targeting ability and stability of PSs and co-deliver various anticancer agents to accomplish integrated cancer treatment [19,62]. The hydrophilicity of NPs can enhance PS solubility in water, thus improving its cellular uptake. The enhanced permeability and retention (EPR) refers to a pathophysiological mechanism and phenomenon, wherein macromolecules including NPs accumulate over time in the tumour vascularized area and thus attain targeted delivery and retention of antitumor drugs in tumour tissues [63]. Most of the currently approved nanomedicines for clinical use in the treatment of solid tumours are dependent on the EPR effect. This EPR effect facilitates the penetration of NPs and various other active molecules through leaky vasculature and mediates accumulation in the tumour site. In a study, Miller et al. [64] developed a model therapeutic NP (TNPs) containing a clinically tested polymer platform [(poly(lactic-co-glycolic acid) (PLGA)-b-polyethylene glycol (PEG)] and a fluorescent platinum (IV) pro-drug to mediate safe and effective drug delivery for cancer treatment. The researchers observed that an increased level of TNPs accumulated within tumour-associated macrophages (TAMs), and these TAMs serve as cellular drug depots. Then, TAMs gradually secrete their DNA-damaging platinum payload into neighbouring tumour cells [64].

PSs can be passively targeted to tumours via the improved EPR effect by using NPs [65]. Among NPs, polymeric NPs (PNPs) are rapidly advancing in the field of nanomedicine because of the flexibility in modifying their properties via selection of the type of polymer and method of carrier assembly [66,67]. PNPs offer excellent surface functionalization properties, controllable size, and biocompatibility [68,69]. The combination of PDT with PNPs can markedly reduce side effects and improve therapeutic outcomes [70]. For example, PNPs, lipid nanocarriers, and metallic NPs were reported to successfully deliver PSs with high efficiency and minimum side effects in patients with both non-melanoma and melanoma skin cancers [71]. The conventional preparation methods of PNPs include nanoprecipitation, solvent evaporation, and emulsification, where most of the materials used are biodegradable and non-toxic [72]. The size of PNPs can be precisely regulated via modification of preparation conditions, thus improving the permeability and biodistribution of drugs. Chemical modification of PNP surfaces allows for tailored drug delivery. It has been observed that PS encapsulation within PNPs can enhance accumulation and prevent early degradation in tumour tissues [73]. Such alterations can enable targeted PS delivery while minimizing injury to normal tissues [19]. PLGA is a highly effective biodegradable PNP. The US FDA has approved the use of PLGA-based NPs as a drug delivery system owing to their biocompatibility, low toxicity, and sustained- as well as controlled-release properties [74]. Numerous PLGA-based NPs have been formulated for the efficient transport of drugs to glioblastoma cells, such as cisplatin [75], metformin/irinotecan [76], temozolomide and DNA repair inhibitors [77], doxorubicin [78], and paclitaxel as well as methotrexate [79].

This study seeks to outline recent progress in the combined application of PNPs and PDT for glioblastoma treatment. By evaluating various types of PDT and PNPs, this review emphasizes how these innovative approaches can play an important role in overcoming glioblastoma-associated critical challenges, including BBB and tumour heterogeneity. Furthermore, this review also discusses the challenges and future directions for PNPs and PDT, therefore provides an insight regarding the potential solutions to various problems that are hindering their clinical translation in glioblastoma treatment.

## 2. Principle and Mechanism of Photodynamic Therapy

**Figure 1:**
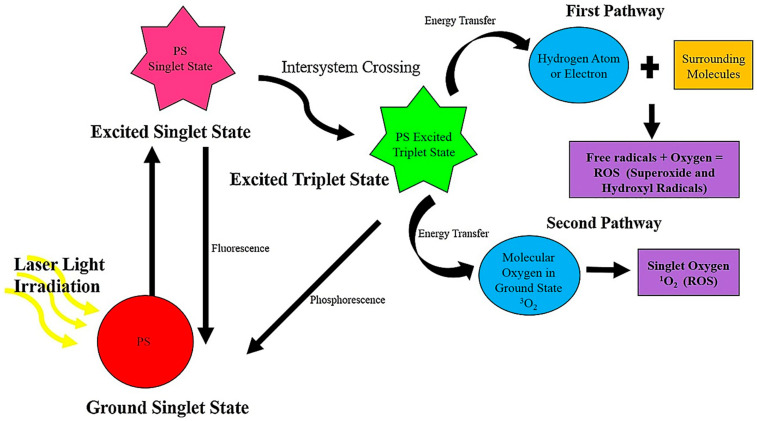
A schematic illustration of the mechanism of PDT. Reproduced with permission from Elsevier, Reference [94]. Following the accumulation of a photosensitizer (PS) in target cancer cells, laser light is used to irradiate the PS. After absorption of energy from the light, the PS agent transits from its ground singlet state to an excited singlet state. On the other hand, most of the energy absorbed by a PS in its excited state is lost through fluorescence, while a small portion of the energy is utilised in the process of intersystem crossing to the excited triplet state. The excited form of the PS in the triplet state can interact with surrounding molecules, including oxygen, to form ROS through two different pathways [86]. The first pathway or type I reaction takes place in the presence of various surrounding biomolecules including lipids, proteins, and nucleic acids within the tumour tissue. The PS molecule in its excited triplet state obtains an electron or a hydrogen atom within the surrounding biomolecules to produce ROS including hydroperoxyl radical HOO^•^, superoxide anion O_2_^•−^, and hydroxyl radical HO^•^. The generated ROS then results in cell damage and the eradication of normal activities via lipid peroxidation. In case of the second pathway or type II reaction, the PS molecule in its excited triplet form transfers energy to ground-state molecular oxygen (^3^O_2_) to yield a highly cytotoxic singlet oxygen (^1^O_2_) [95,96]. In summary, PDT involves the conversion of light energy into toxic ROS [86].

The core principle of PDT relies on the localization of PSs within cancer cells [80]. The first step in PDT involves exciting a neoplastic or inflammatory area with specific wavelength light in the presence of oxygen and PS [81,82]. Then, reactive products including hydroxyl radicals and singlet oxygen are produced at the light-exposed area [83]. The generated free radicals then interact with various molecules including nucleic acids, proteins, peptides, and lipids [84]. They have the capacity to kill cancer cells by stimulating immune responses, damaging tumour blood vessels leading to hypoxia, and inducing necrosis and apoptosis [85]. The group of reactive products in PDT involves the generation of oxygen free radicals mediated by electron transfer (for example, hydroperoxyl radical HOO^•^, hydroxyl radical HO^•^, and superoxide anion O_2_^•−^), and singlet oxygen is obtained through this energy transfer process (Figure 1). Along with oxygen and light, the PS has a crucial role in PDT. PDT encompasses a PS administration that selectively accumulates in the tissues of the patient. A PS molecule is non-toxic and possesses no risk of harmful effects in its inactive form. Nonetheless, irradiated PSs can serve as a source of ROS that can terminate surrounding cells. In this way, PSs will only act in a selective area where it is activated by light, without affecting the healthy tissues. Indeed, singlet oxygen is highly reactive and is capable of damaging DNA and various cellular proteins [86].

PDT is a less invasive therapy that targets all cells or tissues that possess the PS itself, without affecting the surrounding healthy tissues [62,87]. A good PS is highly selective towards the targeted and desired tissues, and shows high cytotoxicity in the presence of light. The type and extent of PDT-mediated damage are largely reliant on the concentration and type of PS used and the wavelength of the light source used [88]. The efficiency of PDT has been clinically proven in the case of superficial lesions on the skin and mucous membranes, thus PDT is already being used in clinics [89,90]. However, deep-seated lesions are a major challenge for PDT owing to the difficult access and limited light penetration [91,92]. Research is ongoing to overcome these limitations [93].

## 3. Polymeric Nanoparticles (PNPs) in the Treatment of Brain Tumours

**Figure 2:**
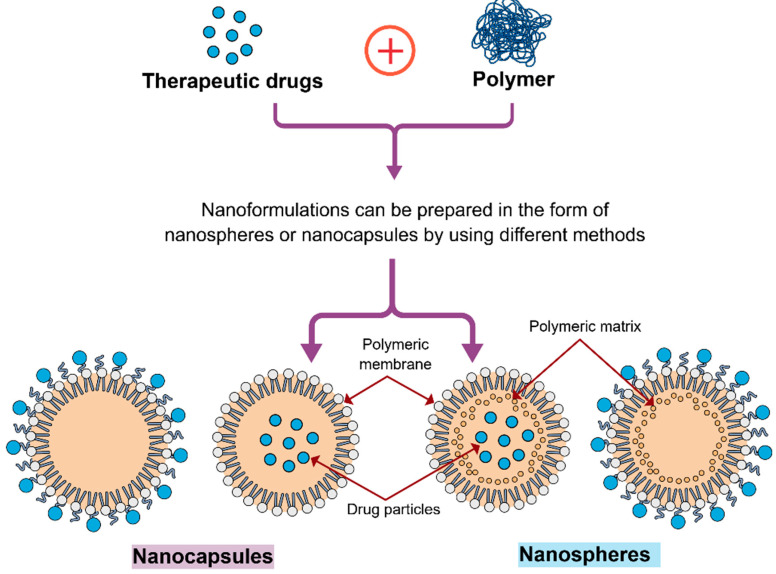
Schematic illustrations of polymeric nanocapsules and nanospheres.

**Figure 3:**
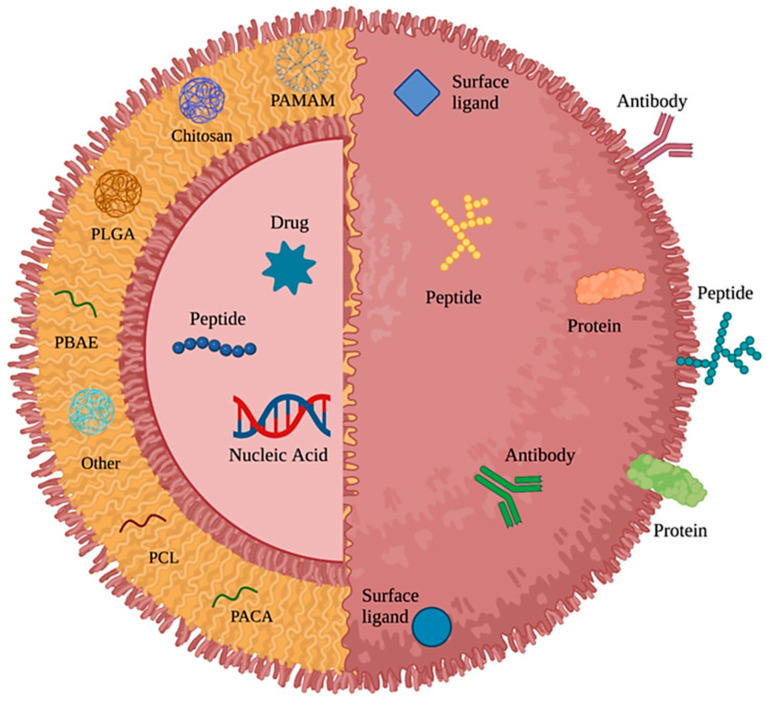
A schematic illustration of the tuneable features of polymeric nanoparticles. This figure is adapted from [106], used under a CC BY 4.0 license. Abbreviations: PAMAM, poly(amidoamine); PLGA, poly(lactic-co-glycolic acid); PBAE, poly(β-amino ester); PCL, poly(caprolactone); PACA, poly(alkyl cyanoacrylate).

PNPs are solid colloidal particles made from biocompatible macromolecular polymers, with a size ranging from 1 to 1000 nm [97]. PNPs can serve as an effective carrier, where drugs or various other active agents are encapsulated, entrapped, dissolved, or adsorbed on the surface of the polymer matrix [98]. Depending on the preparation method, PNPs can form two types of structures including nanospheres and nanocapsules (Figure 2). The BBB provides a major hindrance in the treatment of glioblastoma [99]. A number of biodegradable PNP-based drug delivery systems including dendrimers, chitosan, poly(ε-caprolactone) (PCL), poly(lactic-co-glycolic acid) (PLGA), polylactide (PLA), etc., have already been developed [100,101,102]. Because of their versatile, tuneable features (Figure 3), the PNPs can be designed to open tight junctions of the BBB, easily transport drugs across the BBB, protect against enzymatic degradation, prolong the systemic circulation, and release the drug in a sustainable manner [103]. In a study, Lo et al. [104] developed lipid PNPs modified with tight junction-modulating FD7 and CCD peptides to mediate the delivery of afatinib (an antineoplastic agent) across an in vitro BBB model. The researchers established the in vitro BBB model by growing bEnd.3 cells on Transwell inserts. They observed that CCD and FD7 modulated tight junction proteins (including ZO-1 as well as claudin 5), decreased transendothelial electrical resistance, as well as enhanced the permeability of paracellular markers across the bEnd.3 cells. In addition, the modified or engineered PNPs were partially transported via caveolae- and clathrin-mediated transcytosis, indicating the efficient activation of transcellular and paracellular cascades to mediate afatinib delivery across the BBB [104]. In another study, Wen et al. [105] showed that vascular endothelial growth factor released by glioblastoma cells can have a significant contribution in enhancing the permeability of the BBB via disturbing endothelial tight junction proteins claudin-5. They encapsulated doxorubicin into the hydrophobic core of Angiopep-2-modified glycolipid-like copolymer micelles for efficient entry into the brain tumour region for glioblastoma-targeting therapy. GBM growth leading to BBB pathological fenestration was detected by the researchers both in vivo and in vitro. Indeed, the BBB pathological fenestration in glioblastoma resulted in exposure of more LRP1 binding regions for the doxorubicin-loaded glycolipid-like NPs to the target brain tumour, which further resulted in a stronger brain tumour biodistribution in vivo and a higher transmembrane transport ratio in vitro, and finally exerted substantial antitumor activities [105].

Depending on the nature of loaded drugs as well as their routes of administration, various preparation methods are used for PNPs, including layer-by-layer, reverse salting out, emulsification, nanoprecipitation, solvent diffusion, and solvent evaporation [72,107,108]. The physicochemical features of the drug, and the stability, crystallinity, and molecular weight, can be analysed to develop PNPs for targeting brain tumours [46,72]. Tumours can be targeted by NPs by using both active and passive targeting. Several in vivo studies have already shown that PNPs can circulate for an extended period and selectively accumulate in the tumour site through the EPR effect (so-called “passive tumour-targeting”) [109,110,111]. However, the treatment effectiveness of non-targeted tumour medication delivery systems is suboptimal. A primary reason is the limited tumour cell uptake due to their stealthy surface, which is necessary for prolonged circulation [112]. It has been observed that surface modification of stealth NPs through a selective tumour-homing ligand including folic acid, saccharide, polysaccharide, aptamer, peptide, antibody fragment, antibody, and so on can significantly enhance accumulation and retention of NPs in the tumour vasculature as well as effective and selective internalization via target tumour cells, which is known as “active tumour-targeting” [113]. Both in vitro and in vivo study findings indicate that ligand-directed active targeting NP drug delivery systems show enhanced, though to varying extents, therapeutic outcomes in comparison with their passive targeting counterparts [114,115]. However, it needs to be noted that the development of ligand-mediated active targeting NPs is currently in its infancy. Numerous preclinical studies have been conducted worldwide with different tumour models and NPs, but only a few of the active targeting NPs have advanced to clinical studies [111].

In a study, Vijayakumar et al. [116] reported that resveratrol-loaded PLGA NPs showed marked cytotoxicity and excellent cell internalisation in C6 glioma cells. The resveratrol-loaded PLGA NPs exhibited an extended release pattern without any burst release. The researchers indicated diffusion as the probable drug release mechanism [116]. In addition, a significantly higher level of brain distribution was observed with resveratrol-loaded PLGA NPs as compared to resveratrol solution, which suggests its passive brain targeting potential [116].

In a different study, researchers revealed that docetaxel-loaded NPs showed significantly higher cytotoxicity in comparison with the free docetaxel in the case of glioma treatment [117]. In another study, Kou et al. [118] revealed that L-carnitine-conjugated PLGA NPs showed excellent BBB penetrating capacity and exhibited significant anti-glioma effect. Various recent studies have also indicated the potential of PNPs in improving the delivery of chemotherapeutic drugs to treat glioblastoma. For example, temozolomide is used as a first-line chemotherapeutic agent in glioblastoma treatment, which can be loaded into PNPs to enhance the therapeutic outcome. In a study, Lin et al. [119] developed a polyethyleneimine-based PNP to treat glioblastoma in a mouse model by combining macrophage membrane-coated NPs containing low-frequency ultrasound irradiation.

Glioblastoma cells and brain endothelial cells typically overexpress various receptors that are used as targets for drug delivery in the brain; such receptors include the nicotinic acetylcholine receptor, transferrin receptor (TfR), interleukin (IL)-13 receptor, glucose transporters (particularly GLUT1) and low-density lipoprotein receptor [120,121]. It has been reported that pluronic micelles showed enhanced BBB penetration and demonstrated their capacity to suppress drug efflux [111]. In a study, Sun et al. [122] developed TfR-T12 peptide-modified polyethylene glycol (PEG)-PLA polymeric micelles to deliver paclitaxel against glioblastoma. The researchers observed that the developed polymeric micelles can effectively overcome the BBB and achieve targeted drug delivery in the case of glioblastoma treatment [122]. Table 2 provides a summary of various potential PNPs for the treatment of brain tumours.

**Table 2:** A summary of selected polymeric nanoparticles for the treatment of brain tumours.

Polymeric Nanoparticles (NPs)	Particle Size	Drug/Active Molecule	Targeting Strategy	Study Model	Study Outcome	References
Transferrin-functionalised NPs	137 nm	Temozolomide and the bromodomain inhibitor JQ1	-	Human U87MG and murine GL261 cells	Transferrin-functionalised NPs elevated DNA damage as well as apoptosis that associates with a 1.5- to 2-fold reduction in tumour burden and corresponding improvement in survival	[123]
Chitosan NPs	184.33 ± 4.4 nm	Superparamagnetic iron oxide and doxorubicin (DOX)	-	Rat glioma C6 cells	Chitosan NPs showed potential as an effective theragnostic formulation for both the treatment and diagnosis of glioblastoma	[124]
Hyaluronan (HA)-grafted lipid-based nanoparticles (LNPs)	100 nm	RNA interference (RNAi)	Active	Human glioblastoma U87MG orthotopic xenograft model	In an orthotopic model, mice treated with RNAi-loaded LNPs coated with HA showed markedly improved longevity	[125]
Human serum albumin (HSA)-based NPs	90.5 ± 3.1 nm	Paclitaxel (PTX)	Active	Orthotopic glioma-bearing mice	Improved anti-glioma efficacy was observed with the dual-enhanced system of dual cationic absorptive transcytosis and glucose-transport by the combined usage of c- and m-HSAs	[126]
Albumin NPs	Less than 150 nm	PTX and fenretinide	Active	Human glioma U87, U251 cells, mouse glioma C6, GL261 cells	Albumin NPs showed enhanced blood–brain barrier penetration, intratumoral infiltration, and cellular uptake along with reduced toxic side effects	[127]
Activatable low molecular weight protamine (ALMWP) conjugated with polyethylene glycol (PEG)-polycaprolactone (PCL) NPs	121 nm	PTX	Active	C6 cells implanted into the right striatum of male BALB/c nude mice	Enhanced tumour penetration and glioma-targeting resulted in an anticipated improvement in the in vivo anti-glioblastoma effect; mice treated with ALMWP-NP-PTX showed significantly higher survival	[128]
cRGD-directed, NIR-responsive gold nanorod/PEG-PCL hybrid NPs (cRGD-HNs)	90 nm	DOX	Active	Human glioblastoma U87MG cells	The combined therapy with NIR irradiation and cRGD-HN-DOX completely suppressed tumour growth and showed much lower side effects as compared to free DOX	[129]
PCL NPs	202.1 ± 2.0 nm	Irinotecan hydrochloride trihydrate (IRH)	Active	Primary high-grade glioma (HGG) cells	IRH-loaded nanoparticles exhibited higher encapsulation efficiency and showed cellular toxicity against primary glioma cells	[130]
Polysorbate 80 (PS 80)-coated [^14^C]-Poly(butyl cyanoacrylate) NPs	252–257 nm	DOX	-	Glioblastoma 101/8-bearing rats	Improved penetration characteristics were seen as a result of nanoparticles that were localised in close proximity to the tumour	[131]
Transferrin-modified PEG-poly lactic acid NPs	153.3 ± 28.2 nm	Resveratrol	-	C6 and U87 glioma cells	As compared to free resveratrol, resveratrol conjugates markedly reduced tumour volume and buildup in brain tumours, which eventually led to prolonged survival of C6 glioma-bearing rats	[132]
PLGA NPs	74 ± 18 nm	PTX	-	Intracranial tumours in immunocompromised rats by injection of U87MG cells	PTX-loaded NPs enhanced survival in tumour-bearing rats	[133]
Synthetic protein NP (SPNP) based on polymerised HSA	115 ± 23.4 nm	Small interfering RNA	-	GL26 syngeneic mouse glioma model	SPNPs resulted in long-term survival in 87.5% of glioblastoma-bearing mice and primed the immune system to develop immunological memory against glioblastoma	[134]

## 4. The Importance of Polymeric Nanoparticles in Photodynamic Therapy

There are several challenges involved with conventional PDT, including biodistribution of the PS in the targeted area, oxygen consumption during PDT, oxygen and PS reliance, light penetration depth in tissues, and persistent eye and skin photosensitivity [135,136,137,138,139]. Thus, various efforts were made to enhance penetration depth for deep tissue treatment, regulate the biodistribution of PSs, and ameliorate the oxygen supply of the tumour tissues [140,141]. PNPs were widely investigated by researchers for enhancing the selective targeting of PDT. Various physicochemical features of PNPs including biodegradability, biocompatibility, surface charge, as well as the small size of PNPs make them competent delivery systems for targeting therapy through accumulation at the infarcted sites and targeting specific receptors [142]. It has been observed that the surface charge of NPs has a substantial contribution to the transport of PSs to their targeted areas, thus boosting their clinical effectiveness [143]. Since the cell membrane is negatively charged, the buildup of positively charged NPs within the cell membrane can be effectively attained via wrapping the NPs with a nonantigenic, positively charged polymer. It was reported that PDT improved the internalization of these NPs into the cell membranes, which further improved their clinical effectiveness [142,143].

## 5. Applications of Polymeric Nanoparticle-Based Photodynamic Therapy in the Treatment of Glioblastoma

### 5.1. Photodynamic Therapy with Conjugated Polymer Nanoparticles

Conjugated polymers (CPs) and NPs made of conjugated polymers (CPNs) have great potential in PDT because of their unique biocompatibility as well as optical properties [144,145]. CPs show effective energy transfer abilities and strong light absorption, which are important for ROS generation following light activation [146]. CPNs can improve PS delivery directly into tumour sites, which results in enhanced therapeutic efficiency while reducing injury to surrounding healthy tissues [147]. Furthermore, the capacity to functionalize CPNs with targeting ligands mediates selective buildup in cancer cells, which makes them a potential tool for localized treatment in several types of cancers, including glioblastoma [148,149].

In a study, Caverzán et al. [150] developed a metallated porphyrin-doped CPN for PDT that efficiently resulted in tumour-specific cell death through photo-induced ROS generation. The CPNs played a role as a densely packed multi-chromophoric system containing excellent (intra-particle) energy transfer and light-harvesting capacities, which resulted in effective photo-induced ROS generation. The researchers compared the anticancer properties in three glioblastoma cell lines (along with different initial redox status) through ROS-induced PDT using CPNs. They observed that T98G cells were the most competent in incorporating NPs; however, these cells showed the most resistance toward CPN-PDT effects. Gene expression analysis revealed that this phenomenon might have occurred because of the basal and PDT-mediated antioxidant enzyme levels present in these cells. Moreover, the status of cell-specific antioxidant enzymes is an important characteristic of glioblastoma heterogeneity, therefore establishing its link with the CPN-PDT outcome may be crucial for the development of improved and novel CPN-based therapies [150].

In a study, Liang et al. [38] developed a targeted PDT based on CP and EGFRvIII for glioblastoma treatment. They used a poly [2-methoxy-5-(2′-ethylhexyloxy)-*p*-phenylenevinylene] core to prepare CPNs modified with anti-EGFRvIII (PPVN-A) that showed increased ROS-generating capacity upon white light irradiation. The researchers observed that PPVN-A targeted EGFRvIII-overexpressed tumour cells and caused damage in over 90% of tumour cells upon light irradiation, whereas PPVN without alteration did not exert any obvious cytotoxicity in these cells under the same conditions. Collectively, these findings indicate great potential for PPVN-A as a PDT-based treatment against glioblastoma [38].

In a different study, Ibarra et al. [151] evaluated monocyte-based delivery of CPNs to enhance PDT in glioblastoma. The researchers used murine monocytes isolated from bone marrow (mBMDMs) and human monocyte cells as stealth CPN carriers for effective penetration into an orthotopic model of the tumour and glioblastoma spheroids. They observed that monocyte viability was not affected by CPNs in the absence of light and did not exhibit nonspecific secretion following cell loading. As compared to the monocytes in their naive state, activated monocytes incorporated CPNs to a greater extent without losing cellular functions. The efficacy of PDT in vitro utilizing cell-mediated delivery was found to be superior than utilizing non-vehiculized CPNs. Collectively, these findings suggest that CPN-loaded monocytes might effectively deliver CPNs into the orthotopic model and glioblastoma spheroids, which further indicates the potential of CPN delivery and PDT in glioblastoma treatment.

In another study, Arias-Ramos et al. [152] developed metallated porphyrin-doped CPNs for highly effective PDT to treat glioblastoma. They designed and developed the CPNs via incorporating a metal oxide magnetic core into their matrix during the nanoprecipitation method. The researchers observed that this modification mediated in vivo monitoring of NPs in animal models by utilizing intravital fluorescence and magnetic resonance imaging (MRI) for the evaluation of intracranial tumours. The developed CPNs were evaluated in glioblastoma-bearing mouse models, both orthotopically and heterotopically developed models. They performed biodistribution studies by using fluorescence images and MRI acquisitions up to 24 h after intravenous administration of the NPs. It was observed that the iron oxide NP (IONP)-doped CPNs showed in vitro biocompatibility in glioblastoma tumour cells along with an outstanding cell incorporation based on NP concentration exposure. Furthermore, the IONP-doped CPNs were identified in excretory organs and tumours of the heterotopic glioblastoma model following intratumoral and intravenous injections. Nonetheless, the size of the NPs perhaps impedes a greater effect on intratumorally T2-weighted image signals and T2 values. The IONPs incorporation into the NPs did not affect the PDT-cytotoxicity of CPNs [152].

In a study, Zhang et al. [153] developed a multifunctional, biodegradable CPN containing the PSs 5-ALA and aPDL1. They conjugated the surface of the NP with the ligand of kinin B1 receptor to mediate delivery across the BTB. In addition, 5-ALA was transformed into PpIX upon irradiation with a 980 nm laser, which resulted in the generation of ROS. PDT further mediated intratumoral infiltration of cytotoxic T lymphocytes and sensitized tumours to the PDL1 inhibitor. It was also shown that the combination of aPDL1 and PDT can significantly inhibit the growth of glioblastoma in mice. Collectively, the study findings indicated the potential of CPNs as an effective and novel approach for mediating anti-glioblastoma photoimmunotherapy [153].

### 5.2. Photodynamic Therapy with Poly(Lactic-Co-Glycolic Acid)-Based Nanoparticles

Berberine (BBR) is a naturally occurring isoquinoline alkaloid derived from the *Berberidaceae* plant family [154]. BBR is traditionally used in Chinese medicine and is known to penetrate the BBB, which shows its beneficial effect on the central nervous system (CNS) [148]. Nonetheless, because of the limited solubility of BBR, it suffers from decreased oral bioavailability [155], therefore its overall therapeutic efficacy is relatively low. In order to overcome these limitations, BBR has been loaded into NPs [156]. BBR has also been loaded into PLGA NPs by using the double emulsion method [157].

In a study, Comincini et al. [158] encapsulated two BBR hydrophobic salts including BBR dodecyl sulfate (BBR-S) and BBR laurate (BBR-L) into PLGA-based NPs stabilized with chitosan oleate. In addition, the developed NPs were further functionalized with folic acid. The researchers observed that BBR-loaded NPs were effectively internalized into T98G glioblastoma established cells, and folic acid enhanced this internalization. Nonetheless, without folic acid, the maximum mitochondrial co-localization proportions were achieved with BBR-S NPs. BBR-S NPs were found to be most effective in triggering cytotoxicity in T98G cells, thus they were used to evaluate the activities of PDT. It was observed that PDT induced the reduction of viability for the BBR-S NPs with all studied concentrations, and approximately 50% viability reduction was achieved. No noteworthy cytotoxic activities were observed in normal rat primary astrocytes. On the other hand, a marked rise in late and early apoptotic events was observed in glioblastoma cells by using BBR NPs, which was further increased after the use of PDT. As compared to PDT-only treated and untreated cells, a substantially elevated level of depolarization of mitochondria was observed after the internalization of BBR-S NPs and most commonly after PDT stimulation [158].

Curcumin (CUR) is a bioactive phenolic compound derived from the rhizome of *Curcuma longa* (turmeric), which shows strong anti-inflammatory, wound-healing, and anticarcinogenic properties [159,160]. CUR has recently been identified as a potential PS in antimicrobial PDT [161]. However, CUR shows poor solubility in aqueous solution and poor bioavailability, which has limited its uses in the treatment of several cancer types and various other diseases, thus efficient CUR delivery is crucial in medical fields [162]. Antibody-conjugated biodegradable PNPs were developed to improve the PDT effectiveness of CUR on glioblastoma tumour cells. They observed that in comparison with the CUR-PLGA NPs alone, MAb-CUR-PLGA NPs showed significantly higher photodynamic toxicity in DKMG/EGFRvIII cells [163].

### 5.3. Photodynamic Therapy with Lipid–Polymer Hybrid Nanoparticles

Lipid–polymer hybrid NPs (LPHNs) are novel drug delivery systems that have the capacity to ameliorate the physical stability and biocompatibility of drugs. LPHNs are core–shell nanostructures that are composed of phospholipids and polymers utilized in the development of liposomes and PNPs [164]. Chitosan is an important biocompatible and biodegradable polysaccharide, which can be used to develop LPHNs that can play a role as layer-by-layer NPs of drugs to be transferred into the brain [165]. The lipid NPs offer numerous benefits over other colloidal systems including low toxicity, easy large-scale manufacturing process, directed and controlled drug release, greater loading capacity, and increased drug stability [166].

It has been observed that the lipid NPs matrix of a liquid and solid mixture shows increased loading capacity as compared to solid lipid NPs and liposomes [167,168]. Moreover, scalability can be easily accomplished with lipid NPs. They also lessen the risk of drug expulsion during storage. In lipid NPs, the lipid structure is inserted in a liquid phase, which averts crystallization of solid lipids by mediating a more disordered and less organized internal system in the NPs [169]. Barbosa et al. [170] developed chitosan-coated LPHNs loaded with a PS for glioblastoma PDT. The chitosan-coated lipid NPs showed stable physicochemical properties and exhibited excellent lipid NPs with highly effective encapsulated PS chloro-aluminium phthalocyanine (AlClPc). The researchers observed that LN(AlClPc)Ct0.1% generated ROS in the presence of light and decreased tumour cell viability as well as proliferation. The in vivo study findings suggested that the use of lipid NPs with PDT reduced the total brain tumour area without causing systemic toxicity in mouse models [170].

### 5.4. Metronomic Photodynamic Therapy with Conjugated Polymer Nanoparticles

Metronomic PDT (mPDT) is a type of PDT that triggers death in cancer cells through intermittent continuous irradiation by using a comparatively weak power of light for an extended period [171]. Regimens of mPDT typically involve the administration of drugs for an extended period and/or at non-toxic and low doses, which can lead to prolonged treatment durations as compared to conventional PDT regimens [172]. In terms of PDT, the mPDT regimen involves the administration of low doses of PS and low light irradiance for a prolonged period to enhance the specificity of the therapy and reduce the occurrence of drug resistance. Various molecular PSs including photofrin and 5-ALA were used to assess the effectiveness of mPDT [173,174].

In a study, Caverzán et al. [175] developed a PDT with an advanced PS based on CPN and compared the efficiency of mPDT and conventional PDT involving high light irradiance (fluence rate). The researchers carried out the in vitro assessment based on cell viability, the modulation of hypoxia-inducible factor-1 alpha as an indirect oxygen consumption indicator, and the effect on the macrophage population of the TME in co-culture conditions. As compared to the conventional modality, the mPDT regimens with CPNs led to enhanced cell death in glioblastoma cell lines via different cell death pathways. In addition, the mPDT modality polarized tumour-associated macrophages towards an antitumoral phenotype. In a glioblastoma heterotopic mouse model, CPNs in mPDT markedly slowed tumour growth and mediated cell death along with evident histological alterations [175].

### 5.5. Metronomic Photodynamic Therapy with Polyethylene-Glycolated (Pegylated) Polymeric Nanoparticles

The FDA has approved the use of verteporfin in the treatment of age-associated macular degeneration. Verteporfin can also act as a PS that generates ROS when irradiated with light at 690 nm and can cause localized cytotoxicity [176]. A major benefit of utilizing verteporfin as a PS is its activation at longer wavelengths, which mediates enhanced tumour penetration and ROS generation that results in less peripheral tissue injury [177]. On the other hand, cediranib is a potent inhibitor of vascular endothelial growth factor receptor family tyrosine kinases. In a study, Momeny et al. [178] reported that cediranib decreased glioblastoma cell proliferation, triggered apoptotic cell death, and suppressed the aggressive capacities of glioblastoma cells. Both verteporfin and cediranib show poor water solubility, which affects their bioavailability. Thus, the use of a NP-based drug delivery system can provide protection to the drugs against enzymatic degradation, while delivering a concentrated drug payload to tumour tissues [176]. In addition, NPs with PEG surface modification can escape uptake by the reticular endothelial system, which can further prolong the circulation time of the drug [179]. The EPR effect is an important mechanism for passive tumour targeting, which includes selective accumulation of therapeutic NPs within the tumours because of their unique vasculature [65].

In a study, Kydd et al. [176] designed, synthesized, and evaluated PEGylated polymeric-based verteporfin and cediranib NPs for EPR effects. The researchers observed synergistic effects by using the combined therapies in comparison with the individual drugs. Moreover, they concluded that the use of polymeric encapsulation provided enhanced tumour penetration and subsequent PS cytotoxicity of cancer cells in comparison with the free drug. Moreover, the combined use of cediranib synergistically resulted in increased cytotoxicity of targeted tissues, which may decrease the drug volumes required in glioblastoma treatment.

## 6. Current Challenges and Future Directions

Growing number of studies have already confirmed that glioblastoma cells have the ability to reprogram TME and take over TME elements to mediate rapid proliferation, invasion, migration, as well as their survival, which can further lead to treatment resistance [180]. Clinical trial failure also occurs owing to the heterogeneity of tumour cells between and within patients with glioblastoma [181]. Indeed, PNPs have great potential as NPs; however, various modifications are required in their characterization and synthesis. A major challenge in PNP preparation is their wide distribution of particle size. Furthermore, the diameter of PNPs influences their biodistribution and subsequent efficiency [182]. If a formulation is composed of a wide range of PNP sizes, then its biodistribution and efficiency will be altered. Even though various novel approaches including template-based devices, flash nanoprecipitation, and microfluidic devices are emerging, there is still a need for the development of cheap, novel methods for the preparation of a narrower size distribution of PNPs [183,184]. Various other challenges that need to be addressed include their chemical as well as physical stability, charge and adequate drug loading [185]. The unknown bio-fate of PNPs is another major challenge. Thus, it is crucial to understand the bio-fate of PNPs as well as their pharmacological outcomes, which are needed for their successful translation into patient care [182].

The combination of PNPs and PDT can generate synergistic effects and therefore improve therapeutic efficacy. Although PDT has advanced significantly and provides several benefits over conventional cancer therapies, there are several limitations of PDT that are hindering its translation into clinical use. Such limitations include the dependence of PDT on oxygen levels in the target tissue and tissue penetration depth by light. Other challenges include coping with tumour drug resistance, overcoming uneven drug distribution within tumours, and BBB penetration [186]. These limitations are hindering the use of PDT in glioblastoma therapy in clinical settings. PNPs provide numerous benefits as a delivery system, which have great potential in improving the effectiveness of currently available therapies. Several PSs, PNP matrices, and various other targeting components have been used to generate PNP-based PDT that can ameliorate the effectiveness of PDT. Most of the PNPs exhibit excellent tumour-targeting capacities, which are not attainable with molecular PDT drugs. Still, no approved PNP-based PDT is available for clinical use, even though various in vitro as well as in vivo study outcomes regarding PNP-based PDT are promising, which suggests the need to address several challenges. In this regard, the bioelimination profile has to be carefully considered, since it is important to possess a unique kinetic pattern that is different from presently available chemotherapeutic agents. Furthermore, novel PSs need to be explored that are specific and highly efficient, particularly multifunctional PSs and nano-PSs that can penetrate the BBB, which are likely to offer novel applications for PDT [187]. A new clinical FDA approval procedure is another challenge since NP-based therapies are composed of a range of active ingredients. The aforementioned phenomenon is common to most of the NP-based chemotherapeutic agents. However, several NP-based chemotherapeutic agents have reached clinical settings in cancer treatment [188]. Thus, it is likely that PNP-based PDT will progress towards clinical practice in the near future in the case of glioblastoma treatment.

## 7. Conclusions

Glioblastoma is still one of the most aggressive CNS tumours and currently available conventional therapies most often fail to improve overall survival. In glioblastoma treatment, BBB penetration is currently a major challenge, since present chemotherapeutic drugs cannot effectively penetrate the BBB and reach the tumour cells. Conventional PDT faces challenges in clinical use because of the inadequate accumulation of PSs in the tumours. NP-based delivery systems hold significant promise in transforming cancer therapy, mainly for treatment-resistant and highly aggressive malignancies like glioblastoma. The use of engineered PNPs can be beneficial, as they allow the delivery of a large number of different components for therapy to the targeted area. In addition, the combination of PDT and PNPs can significantly decrease side effects as well as ameliorate therapeutic outcomes.

Thus, innovative approaches including PNPs ought to be considered as they are capable of delivering targeted chemotherapeutic agents to brain tumours. Multiple studies have already shown the efficacy of PDT in improving the median survival of patients with gliomas. Therefore, the combination of PDT and PNPs has the capacity to show enhanced anti-glioblastoma cytotoxicity and can efficiently reduce tumour size in comparison with conventional therapies. Despite promising outcomes reported by a range of studies based on in vitro as well as in vivo evaluation of PDT with various PNPs in preclinical models, these outcomes are yet to be confirmed by clinical trials. Moreover, the discussions and findings that have been presented above ought to be further studied to ameliorate the effectiveness, reliability, and specificity of the combination of PNPs and PDT. Rigorous clinical trials are also required to confirm the efficacy of such combinations so that they can ultimately be used in glioblastoma treatment.

## Data Availability

No new data were created or analyzed in this study. Data sharing is not applicable.
